# Structural differences and adsorption behaviour of alkaline metals doped zinc oxide nanoparticles

**DOI:** 10.1038/s41598-022-06092-z

**Published:** 2022-02-10

**Authors:** Nithyapriya Manivannan, Anna Sycheva, Ferenc Kristály, Gabor Muránszky, Peter Baumli

**Affiliations:** 1 Institute of Physical Metallurgy, Metal Forming and Nanotechnology, University of Miskolc, Miskolc-Egyetemváros, 3515 Hungary; 2 MTA-ME Materials Science Research Group, ELKH, Miskolc, 3515 Hungary; 3 Institute of Mineralogy and Geology, University of Miskolc, Miskolc-Egyetemváros, 3515 Hungary; 4 Institute of Chemistry, University of Miskolc, Miskolc-Egyetemváros, 3515 Hungary

**Keywords:** Chemistry, Engineering, Materials science, Nanoscience and technology

## Abstract

Nanotechnology plays a vital role in all the scientific fields including environmental research due to their surface: volume ratio compared to bulk materials. Recent studies prove their effectiveness as pollutant removal and remediation practices. Zinc oxide (ZnO) nanoparticles a multifunctional material with distinct properties and their doped counterparts were widely being studied in different fields of science. However, its application in environmental waste treatment is starting to gain attention due to its low cost and high productivity. Heavy metal pollution is one of the major pollutants affecting aquatic and terrestrial life forms. Pollution in water bodies has also raised alarming concerns in the past decades. Most of the heavy metals are essential elements in trace amounts and omnipresent in the environment, causing toxicity for living organisms, for instance, nickel. In our work, we analysed the prospect of selective removal of nickel ions by different alkaline metals (K^+^, Rb^+^, and Cs^+^) doped zinc oxide nanoparticles fabricated by different treatment methods (as-prepared and heat-treated). We found morphological variations from flower like to rod like owing to the alkaline cations of  the dopants. In addition, the crystal structure and its different fractions presented amorphous content of the fabricated samples increased from 2 to 10 wt% with respect to the atomic radius of dopant in as-prepared samples and not present in heat-treated samples. We report, how the structure and the sample composition directly affected their adsorption behaviour towards Nickel ions in aqueous solutions based on the micro and nano zincite ratio of the ZnO particles.

## Introduction

Zinc oxide (ZnO) is a semiconductor-based metal oxide in materials world and found potential applications in cosmetics, electronics, and pharmaceutical and rubber industries^1–5^ due to its unique properties. The multifunctional characteristics of ZnO can be improved with help of nanotechnology. Nanotechnology helps us to modify the material properties to introduce new structural, electronic, chemical, or mechanical features. The modification of the size of the particles to nanoscale level, at least in one dimension of less than 100 nm helps to create new materials. Respect to their bulk counterparts nanoscale particles have different surface to volume ratio, interfacial properties, and more^6^. ZnO nanoparticles were being studied for their unique properties and applications. Different synthesis methods such as co-precipitation, sol-gel, hydrothermal, solvothermal, mechanical sintering are widely being studied for better production and quality^7^. In nanoscale, ZnO occurs in different dimensions 1D (e.g., rods, needles, springs), 2D (e.g., sheets, plates), and 3D structures (e.g., flower, snowflakes) leading way to various applications as new materials^2^.

On the other hand, doping a manipulative approach to add impurity or new characteristics by incorporating different elements in their parent compound matrix is widely being studied. Likewise, ZnO nanoparticles doped with transition metals are gaining attention due to their properties and structural integrity^8–11^. Doped ZnO nanostructures have rigid morphology whose structure is made up of several regular phases with geometrically aligned alternative metals and oxide atoms in its axes^12–14^. Zinc oxide nanoparticles as such and their doped counterparts with different doping elements were studied in photocatalysis, battery, sensors, and their magnetic and electrical properties^15–32^. New studies on ZnO tetrapods were also budding due to its application of all benefits on the platform^33^.

Water pollution is one of the major environmental concerns which can affect not only the aquatic life also the terrestrial ones. Heavy metal pollution along with other harmful chemicals provides a major threat. There are several heavy metals in bulk and as well trace amounts in the water bodies being contaminated by power plants, fertilizers, dyeing industries, battery industries, and so on. The use of nanotechnology in filtration process is widely being applied nowadays. Activated carbon sources, biologically modified organisms and polymer materials were used in filtration processes. The use of semiconductor nanoparticles to magnetic nanomaterials doped with transition elements is found to be more suitable for pollutant removal^34–36^. In addition, the identification of heavy metals in aqueous solutions could be a solution for extraction and processing of the contaminants in a more effective way^37–41^. The use of zinc oxide nanoparticles and doped with different elements were starting to be also applied in field of wastewater treatment^41^. Among, various pollutants, Nickel (Ni) an essential trace element for life forms and the most found element is one of the major pollutants in water bodies. Nickel contamination is normally noted in safe limits in most water bodies however,  a slight increase and their toxicity can affect various life forms to greater extent^42^. Also, in terms of environmental and pollution studies, doped ZnO nanoparticles exhibit unique properties, providing adsorption of heavy metals. The favouritism of doping to the adsorption process of certain elements over others has been reported^43–47^.

The need for new materials is raised to quantify the trace amounts of heavy metals present in the aqueous medium over the commercial quantitative tools to identify them from their mass. The identification and removal of heavy metals from the water bodies is one of the major concerns of environmentalists. Even though the question of ZnO adsorption are being investigated^48^, their effects based on structural properties of the material were not widely studied. Different dopant with ZnO was being investigated as mentioned before even for water treatment. However, we could not find studies related to alkaline based doping and their effects on adsorption behaviour towards Ni ions. In a broader sense, selective extraction of Ni was not widely studied like other common pollutants.

In our work, we studied the effects of structural changes in the zinc oxide nanoparticles with different dopants (alkaline earth metals- K^+^, Rb^+^, and Cs^+^) prepared by solvo-thermal method *in-situ*. (The *in-situ* prepared samples K^+^ doped ZnO, Rb^+^ doped ZnO, and Cs^+^ doped ZnO referred to as K:ZnO, Rb:ZnO, and Cs:ZnO, respectively.) The samples were divided into two groups based on synthesis technique as follows 1) as-prepared 2) heat treated. The structural effects of the doping material in the ZnO nanoparticles were characterized for their morphology using scanning electron microscopy (SEM) and crystal structure using X-ray diffraction (XRD). The adsorption characteristics of the ZnO particles for Ni ions were analysed with the help of Atomic Absorption Spectroscopy (AAS). We report the effects of doping in ZnO morphology and how it affected the Ni ions adsorption in an aqueous medium.

## Materials and methods

### Materials

Zinc oxide particles were synthesised using Zinc nitrate hexahydrate (Zn(NO_3_)_2_^.^6H_2_O) (Sigma-Aldrich) and sodium hydroxide (NaOH) (Sigma-Aldrich). Additionally, chlorides of K^+^, Rb^+^ and Cs^+^ (Potassium chloride (KCl) (Fluka), Rubidium chloride (RbCl) and Caesium chloride (CsCl)) were used as doping elements. For the adsorption experiments Nickel (II)-chloride hexahydrate (NiCl_2_^.^6H_2_O) (VWR International Ltd., Hungary) was used to prepare the aqueous medium. All the chemicals used were of analytical grade. Throughout the experiments, distilled water is used and analytical grade acetone and ethanol were used for washing the obtained particles.

### Synthesis of zinc oxide and doped zinc oxide nanoparticles

Zinc oxide particles were synthesized using solvo-thermal method. ZnO nanoparticles were prepared from Zinc nitrate of 0.1 M precursor solution, stirred continuously at 500 rpm. The pH of the solution was adjusted to alkaline range (pH 13) by dropwise addition of 40% sodium hydroxide (approximately 2 ml). They were stirred for 15 min for better dispersion, nucleation, and stability. The mixture was aged, by continuous mixing at 500 rpm, under a higher temperature of 80 °C, further, for four hours. The precipitate (white) obtained was washed three times using acetone, ethanol, and distilled water. After the final washing step, the precipitate was air dried at room temperature until a steady mass of white powdered particles were obtained. Zinc oxide particles (white precipitate) obtained from these steps as such will be mentioned as “as-prepared” samples. A half part of the as-prepared powder was heat treated at 400 °C in a furnace for three hours in air atmosphere, which are mentioned as ‘heat treated’ samples.

For doping, 0.1 M precursor solution of each doping material was prepared from their respective chloride salts (KCl, RbCl, CsCl,). The experimental conditions were slightly modified for doping with respect to our reference^39^. Like ZnO preparation, for doping, equal amounts of precursor solutions (ZnO and dopant) were used for the synthesis in the first step and same procedure was followed as described above.

**Figure 1:**
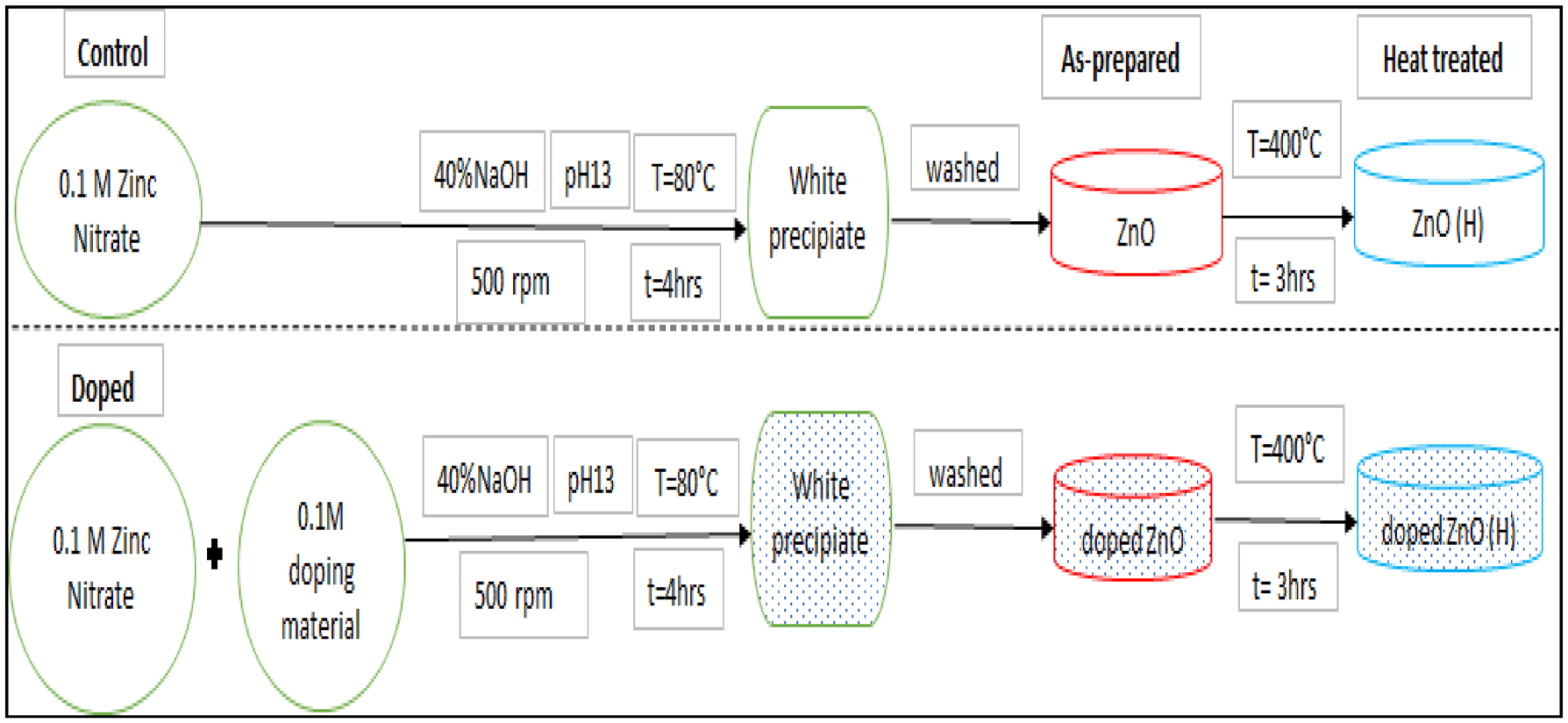
Schematic description of ZnO synthesis and of the doping process.

Generally, the doped ZnO samples were named as K:ZnO, Rb:ZnO, and Cs:ZnO respect to the dopant and sub classified further based as as-prepared (e.g., ZnO, Rb:ZnO) and heat-treated ones(e.g. ZnO(H), Rb:ZnO(H)). The schematic representation with steps for ZnO and doped ZnO particle synthesis is shown in Fig. 1.

### Identification of Ni ions in aqueous solution

Different stock solutions of NiCl_2_ were prepared with the final volume of the Ni solution being 4 ml in concentrations of 30 mg/L to 300 mg/L (30, 60, 90, 120, 180, 210, 300 mg/L). The same amount of the ZnO sample of 25 mg was added to the Ni ion solutions in different concentrations. The mixture was vortexed for two minutes for better dispersion. They were mixed for 60 min (contact time of ZnO particles and the Ni ions) in a horizontal shaker.

Later the suspension was centrifuged, and the supernatant was measured using flame atomic absorption spectrometry (AAS) to control the unabsorbed Ni ions in the solution by ZnO.

## Characterization

### Scanning electron microscopic analysis

The microstructure investigation was carried out using Hitachi S4800 field emission scanning electron microscope (SEM) equipped with Bruker AXS Energy- dispersive X-ray spectrometer (EDS) system. The acceleration voltage of 20 kV was used for the analysis and secondary electron signals was used to study the morphology of the structures at different magnifications (20,000 and 40,000 times).

### X-Ray powder diffraction

The samples were measured by a Bruker D8 Advance diffractometer using Cu Kα radiation (40 kV, 40 mA), in parallel beam geometry obtained with Göbel mirror, equipped with Vantec-1 position sensitive detector (1° window opening). Patterns were recorded in the 2–100° (2θ) angular range, with 0.007° (2θ)/29 s speed. The specimen is rotated in sample plane during the measurement, to obtain data from the whole surface and to reduce in-plane preferred orientation effects. Crystallite size, strain, and unit cell parameters were calculated by Rietveld refinement in TOPAS4, with empirical parametrization, instrumental parameters were defined on SRM 640a Si. Quantitative results were obtained by combined use of Rietveld refinement and peak area calculation (Pawley fit) for the amorphous fraction.

### Flame atomic absorption spectrometry

Pye Unicam (PU 9100) by Phillips was used to measure the Ni content of the aqueous solutions. High purity water (18.3 MΩ/cm) produced by Human corp Zeneer Power I (Scholar-UV) water purification system as well as commercially available 1000 mg/L Ni standard solution were used (Merck, Darmstadt, Germany). Standard solutions were prepared during each batch of measurements. Atomic absorption measurements were carried out using air/acetylene flame. The operating parameters for Ni were set as recommended by the manufacturer. Ni determination was carried out at 341.5 nm absorption line.

## Results and discussion

**Figure 2:**
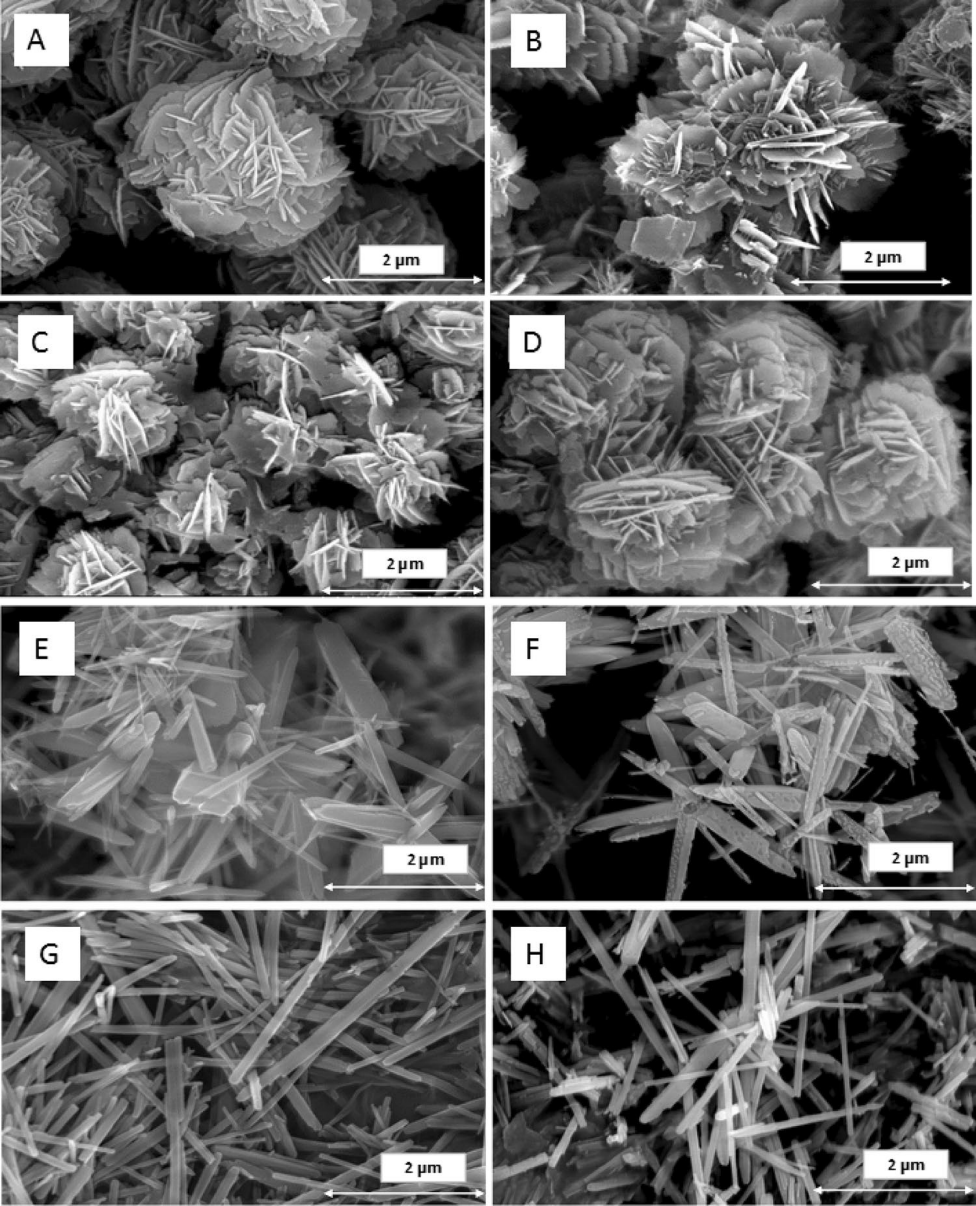
SEM images of as-prepared samples (left) and heat-treated samples (right) before doping (**A**,**B**) and after doping K:ZnO (**C**,**D**), Rb:ZnO (**E**,**F**) and Cs:ZnO (**G**,**H**).

The morphological changes in the ZnO particles based on different doping materials and post heat treatment are shown in Fig. 2. Generally, depending on the doping ions there is a considerable change in the structure of the particles.

The control ZnO samples were flower shaped, K:ZnO particles were also flower shaped (spherical), Rb:ZnO particles were stick like flowery projections and Cs:ZnO particles were sticks stacked together. After heat treatment the samples tend to form rigid morphology and resemble sharp end features. The size of the ZnO and K:ZnO particles visibly increased in size after heat treatment (refer Fig. 2A-D) whereas they decreased in the case of other two doping ions.

The morphological change of ZnO from flower-like to rod-like could be viewed as a function of the atomic mass of the dopant cation. Further changes could be caused by difference in surface energy^49^ of K^+^, Rb^+^, and Cs^+^ ions.

There are different theories (both classical and non-classical) ascribed to the crystal growth of the nanoparticles. In general, synthetic reactions are believed to follow classical models of structural growth, where small nuclei formed from the supersaturated solution, leads to larger particle formation by the initial sacrifice of small particles to a certain extent. It could be also true in our case especially in case of ZnO and K: ZnO where systematic formation is seen at different ranges. On the other hand, ZnO formation is also said to follow non-classical theories, oriented attachment, where larger aggregates are formed and merged in lattices. It could be true in case of lower concentrations and longer duration in the solution matrix leads to the integration of the matrixes. This could be true for the doped particles especially of higher atomic number (Rb^+^ and Cs^+^) as equal amount of precursor elements lower the saturation of the Zn ions in the solution. Also, the stacking or the clustering among the particles is common in particles like ZnO as the system tends to decrease the overall surface energy by matching the crystal lattices by reducing the defects^50^. This also additionally explains the change from flower to sticks with respect to K^+^ to Cs^+^.

The results of EDX analysis were not discussed as they are not feasible to report the atomic mass of dopant present in the samples, due to their less concentration.

**Figure 3:**
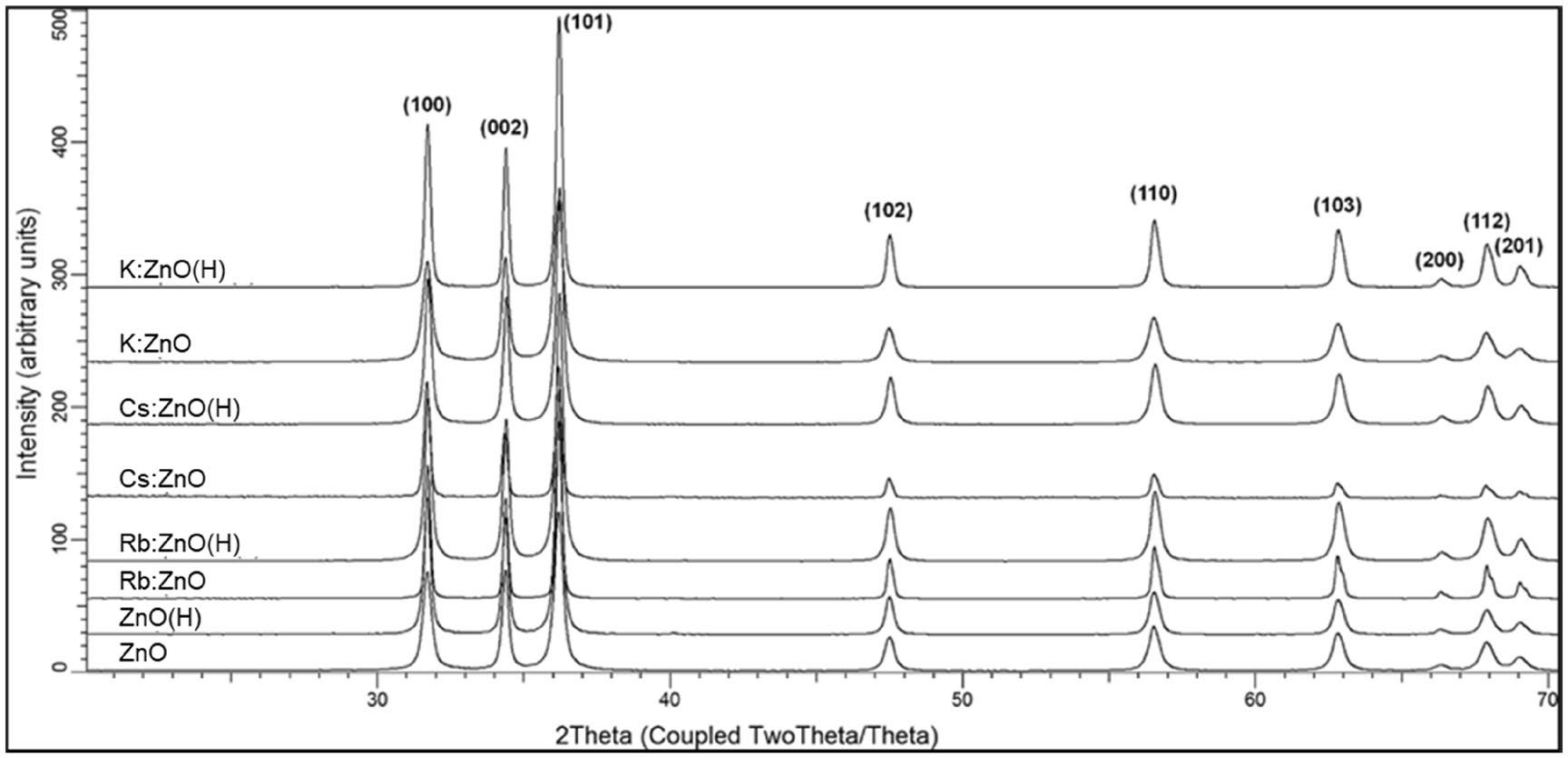
XRD pattern of undoped, doped powders, as-prepared and heat treated ZnO samples.

Structural analysis of the ZnO particles as characterized by the XRD is discussed as follows. A Search/Match algorithm was used to identify crystalline phases, which was only ZnO in all samples (Fig. 3). The differences in peak positions were too small to interpret doping effects on unit cell data. Peak intensities, also a possible data to observe doping effects, were affected by severe and complex preferred orientation, due to the unusual grain morphology. Therefore, Rietveld refinement was used to extract crystal lattice and unit cell data (Table 1).

**Table 1:** Crystallite size information.

Crystallite size (nm)	ZnO	K:ZnO	Rb:ZnO	Cs:ZnO	ZnO (H)	K:ZnO (H)	Rb:ZnO (H)	Cs:ZnO (H)
Micro phase	661 ± 150	890 ± 200	265 ± 100	240 ± 100	890 ± 200	890 ± 200	109 ± 30	364 ± 80
Nano phase	35 ± 10	32 ± 15	133 ± 20	125 ± 20	49 ± 10	73 ± 15	35 ± 10	37 ± 10

**Figure 4:**
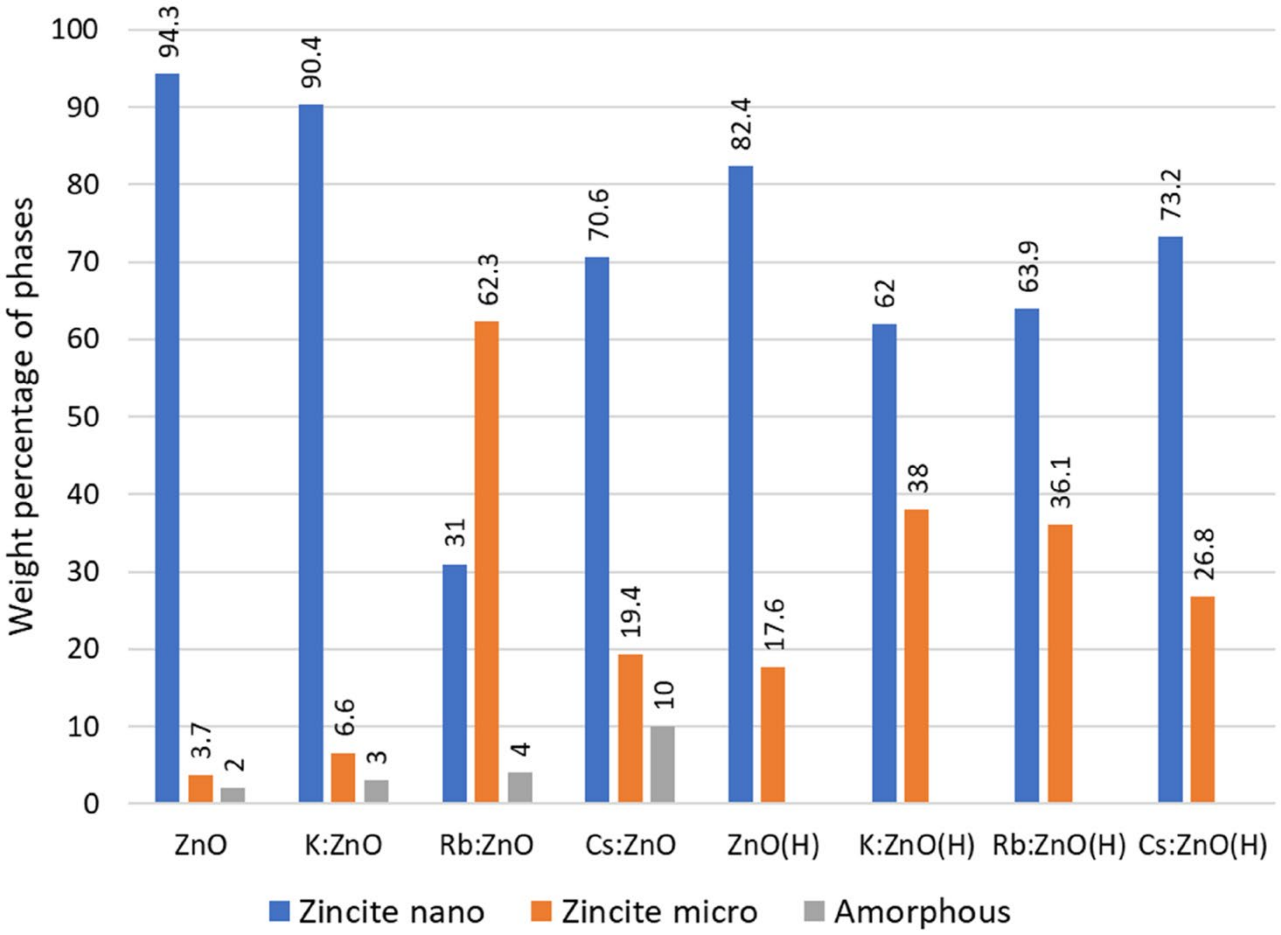
Weight percentage of nano-zincite, micro-zincite and amorphous phases calculated by Reitveld analysis.

**Figure 5:**
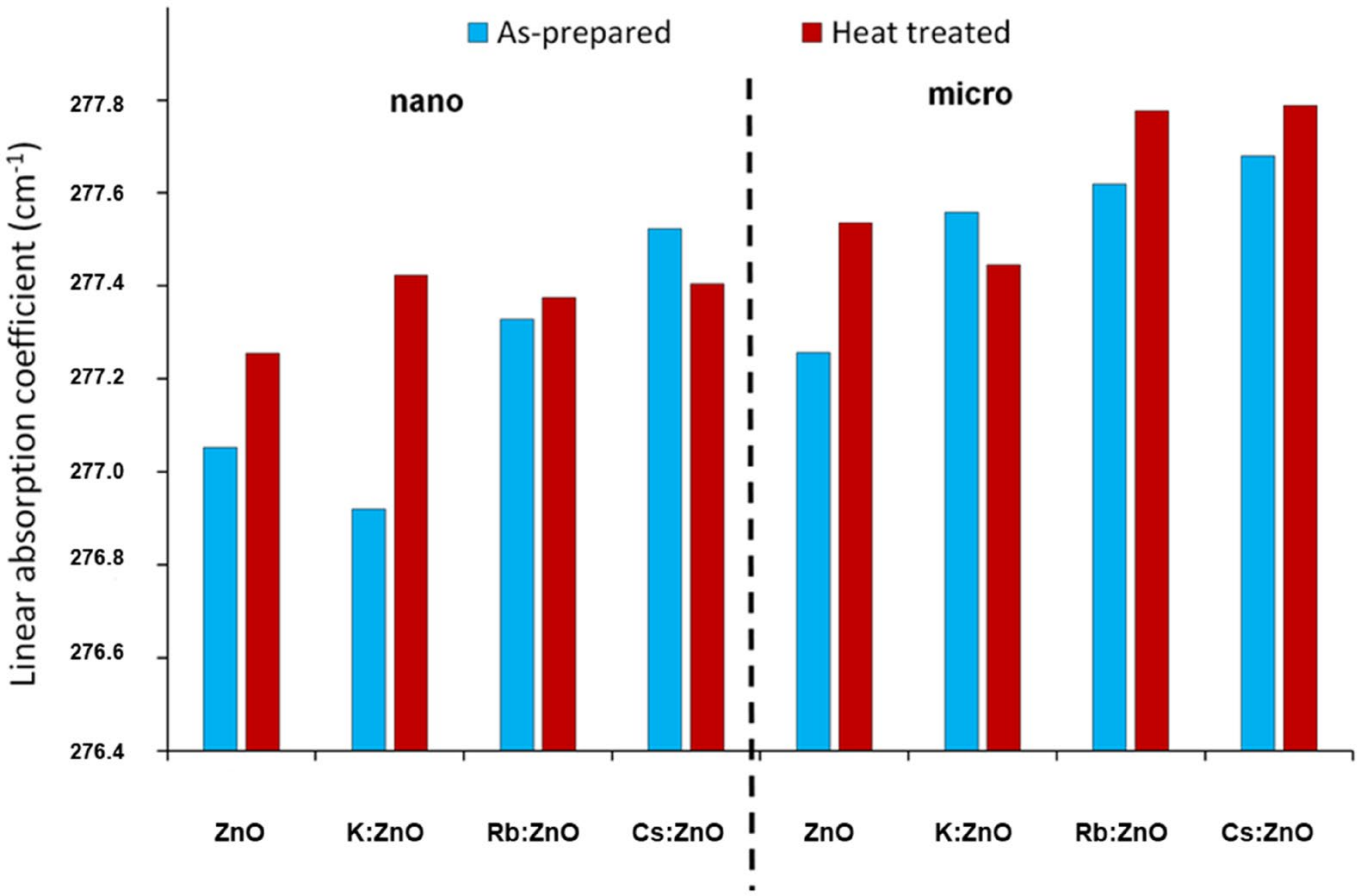
Linear X-ray absorption coefficient of the samples obtained from XRD analysis.

Rietveld refinement also allows quantitative analysis of the samples, in parallel with crystallite size and strain calculation. Preferred orientation was corrected with the March-Dollase function for (001) and (010), but for the most complex particle shapes, we also had to apply the Spherical Harmonic correction, factor 8. With all the corrections and refinements, we observed that none of the samples can be best fitted with one ZnO structure, due to anomalous size broadening. This phenomenon occurs when the crystallite size has a large distribution range, and it is not unimodal. The solution was found by applying one ZnO structure for < 100 nm crystallite size and a second one for > 500 nm sizes. Thus, we categorized the ZnO in two crystallite size regions, as nano and micro range as we consider the range of average size of the particles. This approach allows us to detect the most important size ranges in the distribution, however, offers no information on the size values between the detected ranges (e.g., a distribution of 10 to 1000 nm with a maximum in the 50–100 nm range and a second one in the 600–700 nm does not mean, that all particles are grouped here). Calculating weight percentage offers us a distribution of each size fraction in the sample (Fig. 4), and unit cell data can be refined according to the fractions. Even by this approach, minimal differences were detected between control and doped ZnO unit cell values, indicating an infinitesimal amount of doping cations. However, linear X-ray adsorption coefficient (Fig. 5) shows a characteristic trend according to the atomic weight of the dopant. This property allows us to observe two important properties of the materials: incorporation of heavier or lighter elements by increase or decrease of adsorption coefficient, respectively; presence or elimination of grain boundary porosity/defects, resulting in an increase of adsorption coefficient. The small changes in coefficient of as-prepared samples indicate a < 100 ppm doping cation incorporation. Differences have been observed between nano and microcrystalline fractions for the same samples also. In the case of K:ZnO sample, nanocrystalline fraction takes up all the K^+^, since the coefficient of the microcrystalline fraction is higher than pure ZnO.

After heat treatment generally, a further increase of the coefficient was observed, resulting from the elimination of structural defects^51^, dislocations, and vacancies. These processes occurred simultaneously with nanocrystalline to microcrystalline fraction recrystallization. The highest increase was observed for K:ZnO nanocrystalline fraction, suggesting that by recrystallization K^+^ was incorporated into the microcrystalline fraction, supported by the lower coefficient of heat-treated K:ZnO microcrystalline fraction compared to the same material of pure ZnO.

The presence of hydrozincite was detected for one heat-treated sample (Rb:ZnO) only, but the amorphous phase(s) are present in all as-prepared samples. Some parts of the amorphous fraction could be hydrated, hydrozincite type material. The amount of amorphous fraction increased with the atomic number of the dopant and by heat treatment was crystallized into nanocrystalline doped phases.

Thermal annealing produces changes in unit cell data, crystallite size, and strain also. The presence of amorphous material leads to the formation of the new crystalline fraction during heat treatment, just as observed by SEM. The development of whiskers is possible if an amorphous layer is developed on the surface of the particles during synthesis. Strain is reduced by annealing, indicating the presence of lattice defects in the as-prepared samples which are eliminated by heating. The fraction of micro size range increased by annealing in all samples, except the Rb:ZnO one, which had a higher value of this range in the as-prepared sample. This indicates that Rb^+^ promotes crystallization on ZnO (Fig. 4). The increase in the micro region of samples could take place by sintering and stacking fault elimination during heat treatment.

**Figure 6:**
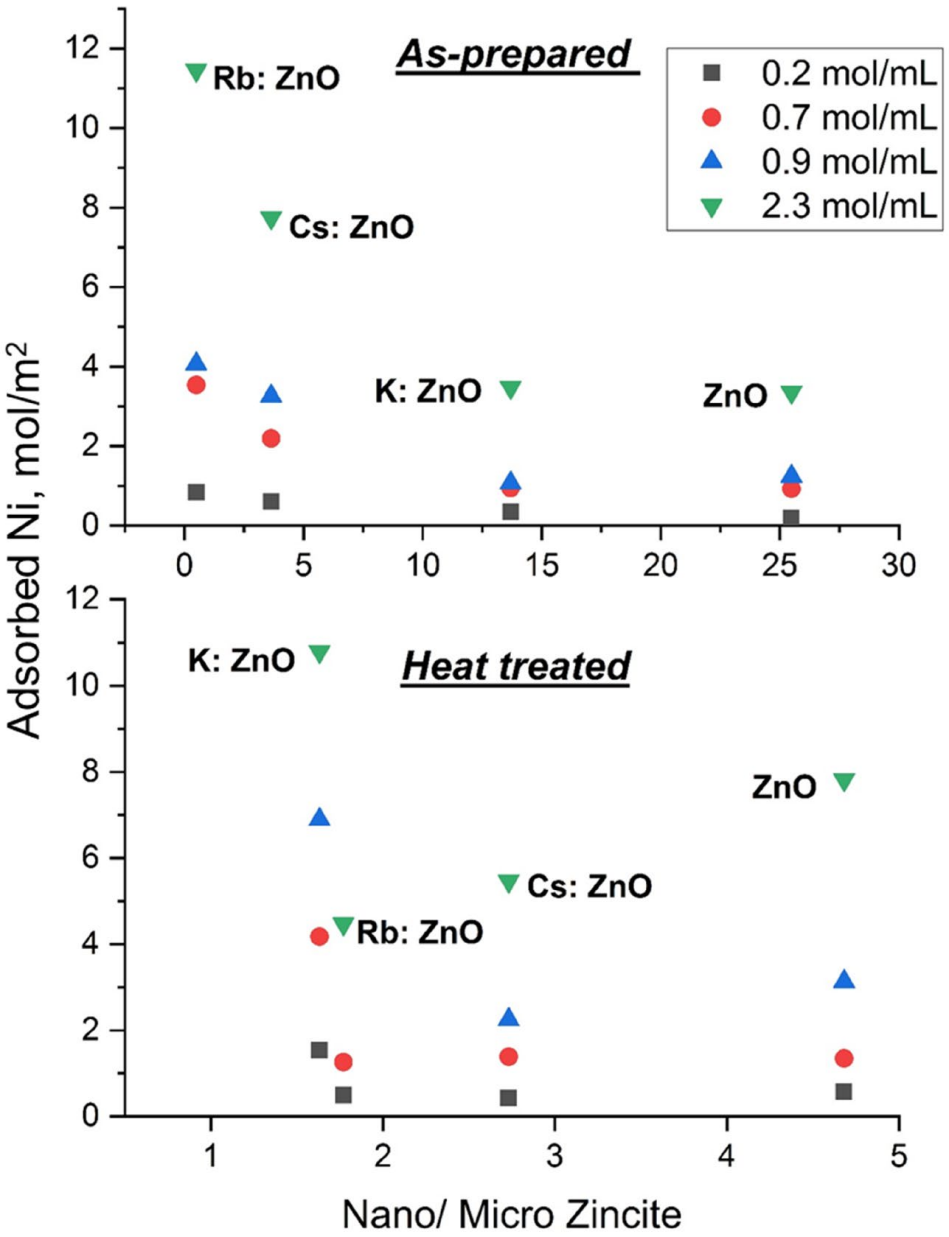
Adsorption of Ni^2+^ ions as function of nano-zincite /micro-zincite ratio of the sample.

The adsorption behaviour of different zinc oxide particles towards Ni ions was plotted using the results from AAS analysis (Fig. 6). In terms of as-prepared zinc oxide particles the adsorption is increased with decreasing the nano-zincite/micro-zincite ratio. That means the higher the micro zincite amount the more Ni^2+^ was adsorbed. The adsorption of the Ni^2+^ on the surface of ZnO particles is influenced by the initial concentration of the Ni^2+^ containing solution. At a higher initial Ni concentration, the amount of adsorbed nickel is higher in the case of as-prepared ZnO, according to Eq. (1):

1 $${\text{C}}_{{{\text{ads}}}} = \left( {4.213{\text{C}}_{{{\text{in}}}} - 0.11} \right){\text{exp}} - 0.05{\upvarphi ,}$$

where C_ads_ is the adsorbed concentration in mol/m^2^, C_in_ is the initial concentration the solution of Ni^2+^ in mol/mL, φ is the nano zincite/micro zincite ratio.

In the case of heat-treated samples, the effect similar to the initial concentration of Ni^2+^ containing solution was exhibited. It means at a higher initial concentration the amount of the adsorbed Ni is higher according to the equation. The adsorbed Ni^2+^ amount is decreased with decrease in the nano-zincite/micro-zincite ratio, in the following order: undoped ZnO, Cs:ZnO, and Rb:ZnO. The lowest amount of Ni^2+^ can be adsorbed on the surface of the heattreated Rb:ZnO. During the heat treating the samples lose the -OH groups from their surface. It is assumed that the lost –OH group amount is increasing in the following order: undoped ZnO, Cs:ZnO, and Rb:ZnO.

## Conclusions

Zinc oxide particles with and without doping were synthesized and characterized specifically for the Ni ions removal in aqueous solutions. K^+^, Rb^+^ and Cs^+^ alkaline earth materials of increasing atomic number were chosen as the dopants for the study.

The microstructure of ZnO transited from flower-like to rod-like as a function of the atomic mass of the dopant cation and caused by the differences in surface energy of K^+^, Rb^+^, and Cs^+^ ions. The crystal growth factors are expected to follow a combination of both classical and non-classical theory based on the solution concentration and surface energy of the alkaline materials.

The inclusion of dopants and heat treatment affects the crystalline fractions of the ZnO particles. The hydrozincite phase is seen only in Rb:ZnO and not in other dopants in case of as prepared samples. It could be the cause of higher adsorption of Rb:ZnO in as-prepared samples at lower Ni concentrations. Also, the amorphous content in the as-prepared samples was increased from 2 to 10 wt% relative to the atomic number of the doping material for pure ZnO and Cs:ZnO, respectively. The adsorption of Ni ions by as-prepared ZnO samples has increased trend based on the nano-zincite/micro-zincite ratio of the samples obtained by Reitveld analysis. In the case of heat-treated samples, these were recrystallized into nanocrystalline fraction of the particles. Leading to more homogenous surface and lesser micro- to nano-zincite ratio.

In terms of as-prepared zinc oxide particles the adsorption is increased with decreasing the nano-zincite/micro-zincite ratio. That means the higher the micro-zincite amount the more Ni^2+^ was adsorbed. The adsorption of the Ni^2+^ on the surface of ZnO particles is influenced by the initial concentration of the Ni^2+^ containing solution. Rb:ZnO sample has the lower nano-zincite/micro-zincite ratio and this sample can absorb the higher amount of Ni ion from the Ni^2+^ containing solution.

In contrary, the samples after heat treatment showed the best adsorption behaviour in the case of K:ZnO samples, their nano-zincite/micro-zincite ratio is lower. The adsorbed Ni^2+^ amount is decreased with decreasing the nano-zincite/micro-zincite ratio, in the following order: undoped ZnO, Cs:ZnO, and Rb:ZnO. This behaviour is assumed to the loosing of the OH groups during the heat treatment.

Thus, our study allows us to understand the effects of different dopants and treatment methods for the better removal of Ni ions. Lower the nano to micro zincite ratio better the adsorption. Also, flower shaped particles have better adsorption over sticks (Cs^+^ doped) in both cases. The results concluded are specific for Ni ions, the trend may vary for different pollutants depending upon their interaction and adsorption trend. Further, our results will help us to formulate precise particles, study different adsorption isotherm models and fabricate a better solution for water treatment.
